# Supplementary material for: Conditional chemoconnectomics (cCCTomics) as a strategy for efficient and conditional targeting of chemical transmission
Source: eLife. 2024 Apr 30;12:RP91927. doi: 10.7554/eLife.91927 (PMC11060718; doi:10.7554/eLife.91927)
Supplement: Figure 3—source data 3. [file elife-91927-fig3-data3.zip › Figure 3 source data3 part 2.zip!/Figure 3-Source data 3-part2/1053f3-1-CRISPResso_Report_t6djfp6u.zip!/CRISPResso_on_1053f3_4/3b.Insertion_deletion_substitutions_size_hist.pdf]

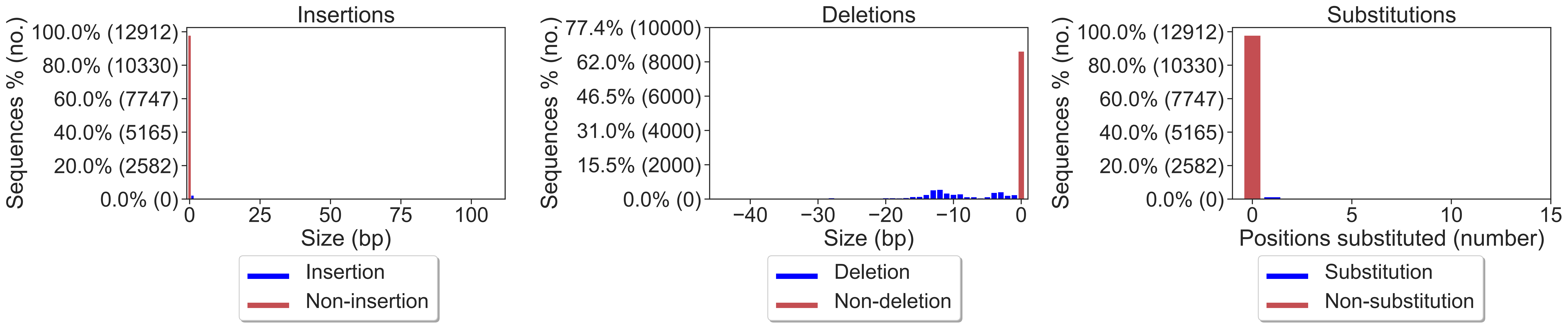

Insertions
Sequences % (no.)
100.0% (12912)
80.0% (10330)
60.0% (7747)
40.0% (5165)
20.0% (2582)
0.0% (0)
0
25
50
75
100
Size (bp)
Insertion
Non-insertion
Deletions
77.4% (10000)
62.0% (8000)
46.5% (6000)
31.0% (4000)
15.5% (2000)
0.0% (0)
−40
−30
−20
−10
0
Size (bp)
Deletion
Non-deletion
Substitutions
0
5
10
15
Positions substituted (number)
Substitution
Non-substitution
